# An evaluation of psychological distress and social support of survivors and contacts of Ebola virus disease infection and their relatives in Lagos, Nigeria: a cross sectional study − 2014

**DOI:** 10.1186/s12889-015-2167-6

**Published:** 2015-08-27

**Authors:** Abdulaziz Mohammed, Taiwo Lateef Sheikh, Saheed Gidado, Gabriele Poggensee, Patrick Nguku, Adebola Olayinka, Chima Ohuabunwo, Ndadilnasiya Waziri, Faisal Shuaib, Joseph Adeyemi, Ogbonna Uzoma, Abubakar Ahmed, Funmi Doherty, Sarah Beysolow Nyanti, Charles Kyalo Nzuki, Abdulsalami Nasidi, Akin Oyemakinde, Olukayode Oguntimehin, Ismail Adeshina Abdus-salam, Reginald O. Obiako

**Affiliations:** Department of Clinical Services, Federal Neuropsychiatric Hospital Kaduna, Barnawa, Kaduna, Kaduna State Nigeria; Department of Psychiatry, Ahmadu Bello University, Zaria, Kaduna State Nigeria; Nigerian Field Epidemiology and Laboratory Training Program (NFELTP), Abuja, Nigeria; Department of Microbiology, Ahmadu Bello University, Zaria, Kaduna Nigeria; Nigerian Stop Polio Program (NSTOP), Abuja, Nigeria; Federal Ministry of Health, Abuja, Nigeria; Department of Psychiatry, University of Lagos, Lagos, Nigeria; Department of Medical Social Services, Lagos University Teaching Hospital, Lagos, Nigeria; United Nation Children Fund (UNICEF), Lagos, Nigeria; Nigerian Center for Disease Control, Abuja, Nigeria; Lagos State Ministry of Health, Lagos, Nigeria; Department of Medicine, Ahmadu Bello University, Zaria, Nigeria

## Abstract

**Background:**

By September 2014, an outbreak of Ebola Viral Disease (EVD) in West African countries of Guinea, Liberia, Sierra Leone, Senegal and Nigeria, had recorded over 4500 and 2200 probable or confirmed cases and deaths respectively. EVD, an emerging infectious disease, can create fear and panic among patients, contacts and relatives, which could be a risk factor for psychological distress. Psychological distress among this subgroup could have public health implication for control of EVD, because of potential effects on patient management and contact tracing. We determined the Prevalence, pattern and factors associated with psychological distress among survivors and contacts of EVD and their relatives.

**Methods:**

In a descriptive cross sectional study, we used General Health Questionnaire to assess psychological distress and Oslo Social Support Scale to assess social support among 117 participants who survived EVD, listed as EVD contacts or their relatives at Ebola Emergency Operation Center in Lagos, Nigeria. Factors associated with psychological distress were determined using chi square/odds ratio and adjusted odds ratio.

**Results:**

The mean age and standard deviation of participants was 34 +/ - 9.6 years. Of 117 participants, 78 (66.7 %) were females, 77 (65.8 %) had a tertiary education and 45 (38.5 %) were health workers. Most frequently occurring psychological distress were inability to concentrate (37.6 %) and loss of sleep over worry (33.3 %). Losing a relation to EVD outbreak (OR = 6.0, 95 % CI, 1.2–32.9) was significantly associated with feeling unhappy or depressed while being a health worker was protective (OR = 0.4, 95 % CI, 0.2–0.9). Adjusted Odds Ratio (AOR) showed losing a relation (AOR = 5.7, 95 % CI, 1.2–28.0) was a predictor of “feeling unhappy or depressed”, loss of a relation (AOR = 10.1, 95 % CI, 1.7–60.7) was a predictor of inability to concentrate.

**Conclusions:**

Survivors and contacts of EVD and their relations develop psychological distress. Development of psychological distress could be predicted by loss of family member. It is recommended that psychiatrists and other mental health specialists be part of case management teams. The clinical teams managing EVD patients should be trained on recognition of common psychological distress among patients. A mental health specialist should review contacts being monitored for EVD for psychological distress or disorders.

## Background

The West African outbreak of Ebola Virus Disease (EVD) began in Guinea in December 2013 [1]. The outbreak involved sustained transmission in Guinea, Liberia, and Sierra Leone [2]. By September 14, 2014, the total number of probable and confirmed cases was 4507, with 2296 deaths recorded from five countries in West Africa namely, Guinea, Liberia, Nigeria, Senegal, and Sierra Leone [3]. The first known case of EVD was reported in Nigeria on 20^th^ July 2014, through a man who travelled to Lagos, Nigeria, via Lomé, Togo and Accra, Ghana [4]. As of 17^th^ September 2014, the total number of confirmed EVD cases in Nigeria was 19 (15 in Lagos and 4 in Port Harcourt) of which 12 had survived and seven reported dead. Four hundred and seventy contacts had completed 21 days of follow up necessary to rule out EVD infection. The news of EVD spread into Nigeria created widespread media attention, which initially focused mainly on the high infectivity and case fatality, with the potential to create fear and panic. Also, the process of infection control and prevention necessary for the control of Emerging Infectious Disease (EID) like EVD involves the use of personal protective equipment, quarantine, and isolation [5], all of which may be associated with fear and anxiety. Public apprehension of newly detected emerging infectious disease with high morbidity and mortality had been previously described. Joffe et al. [6] described pattern of public response to emerging infectious diseases like EVD. This general public response pattern includes distancing the disease from self, blame of particular entities for the disease’s origin and/or spread, and stigmatization of those who have contracted it and/or who are represented as having intensified its spread. The process may be driven by worry, fear and anxiety, which necessitate a psychosocial intervention as part of all outbreak response to EIDs like EVD. In a study to assess the psychological impact of the 2003 outbreak of severe acute respiratory syndrome on hospital employees, about 10 % of the respondents had experienced high levels of post-traumatic stress symptoms [7]. Researches conducted during EVD outbreaks tend to focus on clinical manifestations and epidemiology of EVD with little or no study on psychosocial impact or distress associated with EVD. A study in Democratic Republic of Congo described the feelings and experiences of survivors of Ebola epidemic [8]. They described psychosocial consequences among survivors to include fear of falling seriously ill, denial, fear of being accused by neighbors and shame. Others included rejection by society, belief that the infection was a divine punishment, lack of income, and intense grief for colleagues who did not survive the epidemic. The previous study did not include use of a standardized instrument for evaluation of psychological distress and social support available to the respondents. No previous study that employed the use of standardized instruments like GHQ or OSS to measure psychological distress or social support among survivors and contacts of EVD or their relatives was found after a literature review. We set out to determine the prevalence and pattern of psychological distress among the survivors, relatives and contacts of EVD. We also assessed the social support available for the survivors, relatives and contacts of EVD. Finally, we determined factors associated with psychological distress.

## Method

### Study setting

The study was conducted in Lagos State. Lagos State is located in the Southwestern part of Nigeria and has an estimated land Area of 3474 km^2^ (1341 sq ml). The metropolitan area consists of islands, such as Lagos Island and extension into the adjacent mainland. The Lagos international airport is the busiest of all the international airports in Nigeria [9].

### Study design

We conducted a descriptive cross sectional study.

### Study population

The study population consisted of persons listed as survivors and contacts by the Emergency Operations Center (EOC) for EVD in Lagos and a first-degree relation judged to be the primary care giver by the EVD patient or contact.

### Inclusion criteria

#### Survivors

1. Persons confirmed as a case of EVD in the present outbreak response and had been managed in the isolation ward of the response.
2. Persons confirmed cured and discharged from isolation ward by case management team.

#### Contacts

1. Persons determined by the contact tracing team to have been a contact of a known confirmed case of EVD using standard protocol [10].
2. Contacts who are being actively followed up or had completed the follow up period.

#### Relations of survivors and contacts

1. Must be a first-degree relation (Father, mother, spouse, child or sibling) who was adjudged to have actively supported the survivor during case management or contacts during contact tracing.

### Exclusion criteria

1. Not currently living in Lagos.

### Sample size determination

We estimated that by interviewing a third of survivors and contacts in the line list of the contact tracing team as at the time of the evaluation and their relatives, we will be able to achieve the minimum sample size of 107 calculated using the Leslie and Kish formula [11] for estimating sample size for cross-sectional study.

$$ n = \left({\mathrm{Z}\upalpha}^2\mathrm{p}\mathrm{q}\right)/{\mathrm{d}}^2 $$

Where: *n* = Minimum sample size

Zα set at 5 % significant level = 1.96

p = estimates of proportion of study population with psychological distress. We used the prevalence of 10 % (0.1) psychological consequences of severe acute respiratory syndrome (Similar EID to EVD) among hospital workers in China.

$$ \begin{array}{l}\mathrm{d} = \mathrm{level}\ \mathrm{of}\ \mathrm{p}\mathrm{recision}\ \left(5\%\right)\\ {}\mathrm{q} = 1 - \mathrm{p} = 0.5\end{array} $$

The calculated sample size was 138.

The calculated sample size was adjusted for small population size (*N* = 482) using the formula for finite population correction.

$$ \mathrm{n}\mathrm{f} = \mathrm{n}/1+\left(\mathrm{n}\right)/\left(\mathrm{N}\right) $$

Where:

n_f_ = the desired sample size when population is less than 10,000

n = the desired sample size when the population is more than 10,000

N = the estimate of the EVD survivors and contact or their relations (482) as at the time of study

$$ \begin{array}{l}{\mathrm{n}}_{\mathrm{f}}=\kern1.62em \underline {138\kern2.74em }\hfill \\ {}\kern3em 1+\left(\underline{138}\right)\hfill \\ {}\kern5em (482)\hfill \\ {}{\mathrm{n}}_{\mathrm{f}}=\kern0.5em 107\hfill \end{array} $$

For this study we targeted 120 respondents

### Sampling technique

We randomly selected the contacts for the study using the contact tracing team line list of all contacts with over 200 people listed during the duration of the study. Of the eight cases listed as survivors during the time of study, four were interviewed during the study period. For every contact or survivor selected for the study, we attempted to interview a first-degree relation (spouse, parent, child or full sibling) identified by the survivor/contact, if available and also meeting the case definition for a relation.

### Study instruments

We designed a socio-demographic questionnaire to collect information on the respondents’ age, gender, marital status, Local Government Area (LGA) of residence, and level of education. We also asked if respondents had loss a relation due to the EVD outbreak. The General Health Questionnaire 12-item version (GHQ 12) was used to assess psychological distress among the study participants. The General Health Questionnaire (GHQ) is a screening questionnaire, designed for screening individuals with a diagnosable psychiatric disorder [12]. The GHQ 12 does not generate specific psychiatric diagnosis but rather screens for individuals with possible disorders. In its original version, it had 60 items (GHQ-60), which were reduced to 30 (GHQ-30), and 12 items (GHQ-12) [13]. The 12-Item General Health Questionnaire (GHQ-12) is the most extensively used screening instrument for common mental disorders in addition to being a more general measure of psychological wellbeing. The psychometric properties of GHQ 12 have been evaluated in several studies [14, 15]. The Oslo Social Support Scale (OSS) was used to assess patients’ social support base during the period. The Oslo 3-item Social Support Scale provides a brief measure of social functioning and has been considered a good predictor of mental health [16]. It covers different fields of social support, as it measures the number of people the respondent feels close to, the interest and concern shown by others, and ease of obtaining practical help. The Oslo Social Support Scale had been validated in Nigeria [17]. Respondents who answered “difficult” or “very difficult” to the question “How easy can you get help from neighbors?” were defined as having difficulty getting help from neighbors during need. Respondents who answered “none” or “1–2” to the question “how many people are close to you that you can count on if you have serious problems?” were defined as having less than 3 people who they can count on for help for serious problem. Finally those who answered “no”, “little”, or “uncertain” to the question “how much concern do people show in what you are doing”, were defined as having people showing little concern in what they are doing. Due to the interest in the pattern of psychological distress and social support among the respondents we analyzed each variable in the GHQ 12 and OSS separately instead of using aggregate scores

### Data collection and procedure

We recruited five Resident Doctors of the Nigerian Field Epidemiology and Laboratory Training Program [18] as data collectors, who were part of the contact tracing team and had extensive experience with data collection from prior activities. They were trained for a period of 2 days on the use of the study questionnaires and interview techniques prior to the onset of the study. Data collection took place over a period of 2 weeks and the average duration of each interview was 30 min.

### Data management and analysis

Data were entered into Epi info 3.3.2, cleaned and edited for inconsistencies before analysis. We summarized our findings using frequencies, means (with standard deviation) and proportions. We used Odds Ratio (OR) with 95% Confidence Interval (95 % CI) to check for statistically significant associations and unconditional logistic regression to check for independent predictors of psychological distress.

### Ethical consideration

The evaluation was part of the EVD outbreak response and was therefore exempted from ethical clearance by the EVD emergency operation center in Lagos. The EVD emergency operation center however read and cleared the protocol before onset of the study. Written informed consent was obtained from each participant after complete description of the study. As part of the response to EVD, all the contacts, relatives and survivors who reported or had noticeable distress irrespective of whether they were part of this study or not, had access to counseling and other forms of treatment from the members of the psychosocial subgroup of the clinical management team. Those found to have clinically significant psychological morbidity were counseled and all assessed to require specialist care were referred to the Neuropsychiatric Hospital, Lagos.

## Results

A total of 120 interviews were conducted, of which three were disqualified because the interviews were not completed. The mean age of participants was 34 +/ - 9.6 years, age range 16–62 years.

Of the 117 participants, four (3.4 %) were survivors, 93 (79.5 %) were contacts, 19 (16.2 %) were contact relations and one (0.9 %) was a survivor relation (Table 1). Two thirds of the participants were females and 77 (65.8 %) had a tertiary education. Forty-five (38.5 %) were health workers and about half (48.7 %) resided in Eti-osa Local Government Area (LGA) of Lagos state (Table 1).

**Table 1:** Socio-demographic characteristics of contacts and survivors of Ebola Virus Disease and relations in Lagos, Nigeria, 2014 (*n* = 117)

Characteristics	Number	Percent
Age		
<20	3	2.6
20–49	107	91.4
≥50	7	6.0
Sex		
Female	78	66.7
Males	39	33.3
Religion		
Christians	100	85.5
Muslims	17	14.5
Occupation		
Healthcare workers	45	38.5
Non Healthcare workers	72	61.5
Level of education		
None	2	1.7
Primary	8	6.8
Secondary	30	25.6
Tertiary	77	65.8
Ethnic group		
Yoruba	29	24.8
Ibo	27	23.1
Hausa	4	3.4
Other	57	48.7
Type of respondent		
Survivor	4	3.4
Survivor relation	1	0.9
Contact	93	79.5
Contact relation	19	16.2
Local Government Area		
Eti–Osa	57	48.7
Lagos Mainland	5	4.3
Mushin	34	29.1
Oshodi-Isolo	20	17.1
Surulere	1	0.9

The most frequently occurring psychological distress among all respondents were “Not been able to concentrate on what you are doing” (38.5 %) and “Lost much sleep over worry” (33.3 %). The least occurring psychological distress was “Been thinking of yourself as worthless” (6.0 %). “Not been able to concentrate on what you are doing” and “Lost much sleep over worry” were the most frequently occurring psychological distress among survivors (100 % and 75 %, respectively) and among contacts (35.5 and 33.3 % respectively). Only 22 (18.8 %) of the participants reported “Can count on less than 2 people for help for serious problem” (Table 2).

**Table 2:** Psychological distress/poor social support among contacts and survivors of Ebola Virus Disease and relations, Lagos, Nigeria – 2014 (*n* = 117)

Psychological distress	Survivors	Contacts	Relations	Total
	n (%)	n (%)	n (%)	n (%)
Not been able to concentrate on what you are doing	4 (100.0)	33 (35.5)	8 (40.0)	45 (38.5)
Lost much sleep over worry	3 (75.0)	31 (33.3)	5 (25.0)	39 (33.3)
Been feeling unhappy or depressed	2 (50.0)	25 (26.9)	7 (35.0)	34 (29.0)
Felt constantly under strain	1 (25.0)	24 (25.8)	8 (40.0)	33 (28.2)
Not been able to enjoy your normal day to day activities	1 (25.0)	20 (21.5)	9 (45.0)	30 (25.6)
Not been feeling reasonable happy	1 (25.0)	16 (17.2)	7 (35.0)	24 (20.5)
Felt you are not playing a useful part in things	1 (25.0)	11 (11.8)	3 (15.0)	15 (12.8)
Been losing confidence in your self	0 (0.0)	7 (7.5)	2 (10.0)	9 (7.7)
Felt incapable of making decisions	2 (50.0)	5 (5.4)	2 (10.0)	9 (7.7)
Been able to face up to your problems	1 (25.0)	8 (8.6)	0 (0.0)	9 (7.7)
Felt you couldn’t overcome your difficulties	0 (0.0)	5 (5.4)	2 (10.0)	7 (6.0)
Been thinking of yourself as worthless	0 (0.0)	4 (4.3)	3 (15.0)	7 (6.0)
Social Support				
Can count on less than 3 people for help for serious problem	0 (0.0)	17 (18.3)	5 (25.0)	22 (18.8)
Difficult getting help from neighbors if you need it	1 (25.0)	11 (11.2)	4 (20.0)	16 (14.5)
People show little or no concern in what you are doing	0 (0.0)	6 (6.5)	2 (10.0)	8 (6.8)

Losing a relation to the EVD outbreak (OR = 6.0, 95 % CI, 1.2–32.9) was significantly associated with the psychological distress of “feeling unhappy or depressed” while being a health worker (OR = 0.4, 95 % CI, 0.2–0.9) was protective. Having no tertiary education (OR = 0.3, 95 % CI, 0.1–0.7) was significantly protective against “Not been able to concentrate”, while living in Eti-osa LGA (OR = 3.2, 95 % CI, 1.2–8.5) was significantly associated with “Not feeling reasonable happy”. All the four survivors reported they had, “Not been able to concentrate” (Table 3).

**Table 3:** Associations of psychological distress among contacts/survivors of Ebola Virus Disease and relations, Lagos, Nigeria, 2014 (*n* = 117)

	Yes		No		OR (95 % CI)	*p*
Feeling unhappy/depressed	n	%	N	%		
Lost relation to EVD outbreak	6	66.7	3	33.3	6.0 (1.3–24.4)	<0.01^*^
Been a health worker	8	17.8	37	82.2	0.4 (0.2–0.9)	0.03
Contacts	25	26.9	68	73.1	0.6 (0.2–1.6)	0.31
Relations	7	35.0	13	65.0	1.4 (0.5–3.9)	0.52
Not been able to concentrate						
Having no tertiary education	8	20.0	32	80.0	0.3 (0.1–0.7)	<0.01
Lost relation to EVD outbreak	7	77.8	2	22.2	6.5 (1.3–32.6)	<0.00^*^
Survivor	0	0	4	100.0	-	0.02^*^
Contacts	33	35.5	60	64.5	0.6 (0.2–1.4)	
Not feeling reasonable happy						
Living in Eti-osa	17	29.8	40	70.2	3.2 (1.2–8.5)	0.01
Lost relation to EVD outbreak	5	55.6	4	44.4	5.9 (1.2–29.4)	0.01^*^
Felt not playing useful part in things						
Been female	6	7.7	72	92.3	0.3 (0.1–0.8)	0.02
Not easily getting help from neighbors	6	26.1	17	73.9	3.3 (1.0–12.2)	0.03
Felt constantly under strain						
Living in Eti-osa	23	40.4	34	59.6	3.4 (1.4–8.0)	<0.01
Been able to make decisions						
Not easily getting help from neighbors	5	19.2	21	80.8	5.2 (1.1–25.7)	0.01
Losing confidence in self						
Little or no concern shown by people in your activities	3	37.5	5	62.5	10.3 (1.5–70.8)	<0.01^*^

*Fisher’s exact test

Losing a relation (AOR = 5.7, 95 % CI, 1.2–28.0) remained an independent predictor of the psychological distress of “feeling unhappy or depressed”. Loss of a relation (AOR = 10.0, 95 % CI, 1.7–60.7) remained an independent predictor of the psychological distress of “Not been able to concentrate” while having no tertiary education (AOR = 0.2, 95 % CI, 0.1–0.6) remained a protective factor against “Not been able to concentrate” (Table 4).

**Table 4:** Independent predictors of psychological distress among contacts and survivors of Ebola Virus Disease and their relations using unconditional logistic regression, Lagos, Nigeria – 2014 (*n* = 117)

Term	Feeling depressed AOR	95 % CI		*p*
Lost relation (Yes/No)	5.7	1.2	28.0	0.02
Living in Eti-osa (Yes/No)	2.3	1.0	5.5	0.06
Not health worker/health worker	0.4	0.2	1.2	0.09
Male/Female	1.6	0.6	4.2	0.34
No tertiary education/having tertiary education	1.0	0.4	2.4	0.95
	Inability to concentrate			
No tertiary education/having tertiary education	0.2	0.1	0.6	<0.01
Lost relation (Yes/No)	10.1	1.7	60.7	0.01
Living in Eti-osa (Yes/No)	1.1	0.5	2.5	0.82
Not health worker/health worker	1.2	0.5	2.8	0.65
Male/Female	0.8	0.4	2.0	0.68

## Discussion

The most frequently occurring psychological distress among the respondents: inability to concentrate, losing much sleep over worry and being unhappy or depressed are key clinical features of anxiety, depression and Post Traumatic Stress Disorders (PTSD) as described in the Diagnostic and Statistical Manual (DSM) of mental disorders [19]. Though the individual psychological distress does not amount to a neuropsychiatric disorder, it does indicate the presence of some psychological distress among the respondents that may have the potential to progress if not properly managed. EVD can be perceived as a life-threatening event that meets a key diagnostic criterion of PTSD in DSM [19]. The development of PTSD following life-threatening event has been demonstrated among Nigerians [20] and among health workers who had contact with Severe Acute Respiratory Syndrome (SARS) [7]. Other less severe disorders caused by reaction to extremely stressful situations such as acute stress reaction and adjustment disorders could also present with the above psychological distress. The psychological distress of being unable to concentrate could be mistaken for cognitive impairment by the clinical management team if it occurs in a patient with EVD, could be confused for viral encephalopathy or onset of brain damage secondary to EVD infection. A case of adjustment disorder, in a survivor of the Nigerian EVD outbreak, initially diagnosed as having brain damage secondary to viral encephalitis is an example [21]. Losing a relation during this EVD outbreak was significantly associated with being unhappy or depressed. This could be dismissed as a usual response to bereavement in people, but the feelings of depression (whether from normal grief or psychopathological) could have implication for the management of patients with EVD or for the contact tracing team while monitoring contacts for clinical manifestation of EVD. It could affect judgment and thus reduce cooperation with either the clinical management team or contact tracing team. Feelings of depression could also cause patients or contacts of EVD to tolerate emerging symptoms of EVD thereby not reporting them to the management team. This could complicate overall clinical impression of the patient or cause problems with determining the exact time of onset of clinical symptoms. The effect of an EVD contact, with feelings of depression, failing to disclose important clinical symptoms may lead to delayed or failed recognition of EVD onset with far reaching public health importance. The psychological stress of bereavement can mimic severe depression but also bereavement has long been described as a risk factor for development of depression [22]. Furthermore, it has been suggested that risk factors for common mental health problems arising from the EVD outbreak such as witnessing and caring for individuals who are severely ill, perceived life threat, substantial mortality and bereavement, and the deaths of trained health care workers, in conjunction with the lack of well-trained mental health professionals in countries experiencing EVD outbreak in West Africa could amplify the risks of developing enduring psychological distress and progression to psychopathology by those affected [23]. Although the psychological distress described in this study may not be definitive psychological disorder, there is the need for follow up assessment of the survivors, contacts and relations.

The extensive social support base of the Nigerian community, which is not limited to the immediate family members, may have helped the social support respondents had. Poor social support was only a problem to less than a quarter of the respondents. This may have been due to the relatives not being aware of what the respondents were going through. Overall, only few of the respondents demonstrated poor social support, respondents who had little or no interest shown in their activities were more likely to lack self-confidence. Having no tertiary education, which was protective of inability to concentrate, may not be an entirely positive finding because it could reflect the lack of insight into the implication of an infection with EVD by those without tertiary education. The study found loss of a relation to be an independent predictor of feelings of unhappiness or depression and inability to concentrate. Contacts or survivors who have lost close relations should be considered at high risk of developing psychological distress or even psychological disorders. Loss of a relation is a traumatic experience that has been shown to be a predictor of PTSD [20] and depression [24] among persons exposed to traumatic experience in Nigeria. Therefore the psychosocial response team for EVD outbreak should prioritize this subgroup of contacts and survivors for special monitoring and evaluation. The contact tracing teams following up this subgroup of contacts should include a member of the psychosocial response team with training in detection of psychological distress/disorders.

The findings of this study are subject to the following limitations. We only assessed for psychological distress and not disorders. Only few survivors were sampled which limited the ability to independently look at the dynamics of psychological distress among them. The evaluation could not interview the anticipated number of relatives because some of the contacts did not inform their immediate family members about their status as EVD contacts and therefore could not be interviewed. Despite these limitations, we are confident the findings of this study reflect the possible psychological distress following being a survivor or contact of EVD or a relation to any.

## Conclusions

We concluded that survivors and contacts of EVD or their relatives develop psychological distress that could be predicted by loss of a relation and recommended that mental health specialists and social workers be part of the case management team of the response to EVD outbreak. The clinical teams managing EVD patients should be trained on recognition of psychological distress among the patients and recognition of common psychiatric disorders like depression that could follow EVD infection, and special attention should be paid to those who have lost a relation. We also recommended the follow up of all survivors/contacts with increased risk of developing psychological distress or disorders for a minimum period of 6 months by a mental health specialist for early detection of mental health disorders following EVD. The findings of this study were shared with the Ebola emergency operation center in Lagos and the main findings were equally presented to the meeting of the Association of Psychiatrists and Allied Professionals in Nigeria.
